# CKS1B promotes the progression of hepatocellular carcinoma by activating JAK/STAT3 signal pathway

**DOI:** 10.1080/19768354.2021.1953142

**Published:** 2021-07-14

**Authors:** Xitao Liu, Defang Zhao

**Affiliations:** Department of Hepatobiliary Pancreatic Surgery, Affiliated Hospital of Inner Mongolia Medical University, Mongolia Autonomous Region, Hohhot, People’s Republic of China

**Keywords:** CKS1B, JAK/STAT3 signal pathway, hepatocellular carcinoma, invasion, proliferation

## Abstract

Hepatocellular carcinoma (HCC) is a malignancy of considerable concern due to its continuous increase in morbidity and mortality. This study attempts to identify the molecules that play a key role in the progression of HCC, explore its potential mechanism, and provide more target choices for targeted therapy. Using overexpression plasmid and shRNA, CKS1B was respectively overexpressed and knocked down to explore its biological function roles in HCC progression and development. MTT and colony formation assays showed that knockdown of CKS1B inhibited the survival and proliferation of HCC cell lines (Hep3B and Huh7). The flow cytometry and western blot analysis showed that knockdown of CKS1B significantly induced the apoptosis of Hep3B and Huh7 cells. The wound healing and transwell invasion assays showed that knockdown of CKS1B had a significant inhibitory effect on the migration and invasion of Hep3B and Huh7 cells. These functional tests confirmed that CKS1B acts as an oncogene that regulates the malignant progression of HCC. Moreover, this study also demonstrated that knockdown of CKS1B inhibited the activation of JAK/STAT3 pathway, evidenced by the significantly downregulated p-STAT3 protein expression. Furthermore, knockdown of CKS1B also downregulated STAT3 target genes TIMP-1, Bcl-2 and VEGF, which were involved in controlling cell apoptosis and migration. On the contrary, overexpression of CKS1B caused the completely opposite results. Taken together, CKS1B acts as an oncogene to promote the proliferation and metastasis of HCC cells by activating JAK/STAT3 signaling pathway.

## Introduction

As a well-known heterogeneous malignant tumor, hepatocellular carcinoma (HCC) has seriously damaged human health and wealth. In China, the morbidity and mortality of HCC continue to rise due to high rates in later diagnosis and limited treatment options(Bray et al. 2018; Siegel et al. 2019). In order to inhibit the progression of HCC and improve the survival rate of patients, the treatment strategy for HCC is diversified, including surgical resection, liver transplantation, radiofrequency ablation, sorafenib and other systemic therapy, as well as immunotherapy, cytotoxic chemotherapy, etc. (Liu et al. 2015). The multi-step process involved in the development of cancer begins with a series of genetic and epigenetic events that lead to multiple changes in signaling pathways, such as vascular endothelial growth factor (VEGF) pathway, growth factor pathway, MAPK and JAK/STAT pathway. Targeting these signaling pathways is also considered to be of clinical value in cancer therapy(Lim et al. 2011). Therefore, identification of molecules and related signal pathways involved in the occurrence and development of HCC is of great importance for HCC treatment and prognise.

CKS1B(CDC28 protein kinase regulatory subunit 1B)has been reported overexpressed in breast cancer, lung cancer and other malignant tumors due to the amplification of chromosome 1q21(2012). For example, down-regulation of CKS1B inhibited the proliferation and migration of colorectal cancer cells(Hwang et al. 2019), and also suppressed the invasion and angiogenesis of retinoblastoma cells(Zeng et al. 2019). These evidences suggested that CKS1B may be a key gene to promote the malignant progression in a variety of tumors. As a highly conserved member of the CKS1/suc1 gene family, CKS1B regulates cell cycle process and affects cell proliferation by interacting with cyclin-dependent kinase (CDK) and SCF complex(Bourne et al. 1996; Spruck et al. 2001). In addition, HCC was found to display frequent 1q elevations, and 1q21-22 has been identified as the smallest overlapping region containing candidate oncogenes for HCC (Wong et al. 2003), suggesting that CKS1B is a candidate oncogene for HCC progression. Although CKS1B has been shown to promote the proliferation and invasion of HCC cells by inducing osteopontin (OPN) expression(Kang et al. 2019), its regulatory role in HCC progression still needs to be deeply investigated.

Previous reports have suggested that CKS1B was up-regulated in HCC, and its overexpression was associated with clinical invasiveness, which was regulated by multiple genes and molecular pathways (Okabe et al. 2001; Huang et al. 2010). Among them, the activation of the JAK/STAT3 pathway could induce metastasis and angiogenesis in HCC, and CKS1B overexpression has been previously reported to activate the JAK/STAT3 signaling pathway (Shi et al. 2010; Yan et al. 2017) Therefore, this study attempted to explore whether CKS1B regulates the malignant progression of HCC through JAK/STAT3 to provide a valuable molecular mechanism for targeted therapy of HCC.

## Materials and methods

### Cell culture and transfection

Human HCC cell lines Hep3B (CL-0102) and Huh7 (CL-0120) were purchased from Wuhan Procell Company and identified by STR in advance. The HCC cells were cultured in DMEM growth medium supplemented with 10% FBS and 1% P / S and stored in a 5% CO_2_ wet incubator at 37 °C.

Lipofectamine 2000 (11668027) was purchased from Thermo Fisher Scientific (Shanghai, China) and used for transfection. Short hairpin shRNA and negative control shRNA for knockdown of CKS1B and lentivirus for CKS1B overexpression were purchased from Genetic Chemistry Company (Shanghai, China). The full-length CKS1B sequence was synthesized by RiboBio (Guangzhou, China) and ligated to the pcDNA3.1 plasmid (V79020; Thermo Fisher Scientific). For transfection, cells were mixed with lentivirus and purine toxin was added to the medium after 72 h to select cells infected by lentivirus.

### Western blot analysis

RIPA buffer (P0013 K) was purchased from Beyotime Company (Jiangsu, China) to extract the total protein from HCC cells, and the extracted protein was quantified by BCA method. The sample protein was separated by 10% SDS-PAGE electrophoresis and transferred to PVDF membrane and sealed with 5% skim milk at room temperature for 2 h. After washing, the membranes were incubated with the primary antibodies overnight and then incubated with the secondary antibody Goat anti-rabbit IgG H&L (HRP) (ab205718, 1:5000). Protein bands were observed by an enhanced chemiluminescence (ECL) kit (SW2010, Solarbo, Beijing, China) and Tanon 5200 imaging analysis system (Tanon, Shanghai, China). The antibodies used in this experiment were purchased from Abcam (Cambridge, MA, USA), and the specific information of primary antibodies was shown in Table 1.

**Table 1.** Primary antibody information.

Gene	Item number	Dilution
CKS1B	ab72639	1: 1000
Cleaved Caspase-3	ab214430	1: 5000
Cleaved PARP	ab32561	1: 1000
JAK1	ab133666	1: 1000
*p*-JAK1	ab138005	1: 1000
STAT3	ab68153	1: 1000
*p*-STAT3	ab76315	1: 2000
Mcl-1	ab32087	1: 1000
c-Myc	ab32072	1: 1000
TIMP-1	ab211926	1: 1000
Bcl-2	ab182858	1: 2000
VEGF	ab32152	1: 1000
GAPDH	ab9485	1:2500

### Cell viability assay

Cell activity was analyzed using the MTT kit (M6494, Thermo Fisher Scientific). Cells were inoculated in 96-well plates and placed in an incubator. MTT solution was added to the cells, the medium was discarded and 150 μL DMSO was added to each well. 96-well plates were gently shaken and the absorbance of the samples at 490 nm / 570 nm was measured.

### Colony formation assay

Inoculated 1 × 10^4^ cells in 6-well plates and incubated at room temperature for 2–3 weeks. When colony formation was visible to the naked eye, the culture was terminated. Cells were fixed using 4% paraformaldehyde and stained by 0.1% crystal violet. The plates were washed and dried overnight and colony was counted visually.

### Flow cytometry for apoptosis

According to the instructions, 1  × 10^5^ cells were centrifuged and resuspended. Hep3B and Huh7 cells were stained for FITC Annexin V and PI using the FITC Annexin V Apoptosis Detection Kit (CA1020, Solarbio, Beijing, China). Analysis was performed by flow cytometry within 1 h. Analysis was performed by flow cytometry using an Attune™ flow cytometer (Thermo Fisher Scientific) within 1 h.

### Wound healing experiment

Hep3B and Huh7 cells were inoculated in a 6-well plate to 80% fusion. Discarded DMEM, and used the tip of a 10 μL aseptic pipette to draw in the center of each hole and incubated with FBS-free DMEM for another 24 h. The cell mobility was analyzed by Image J 1.8.0 under inverted microscope.

### Invasion experiment

Hep3B and Huh7 cells were digested with trypsin and suspended in serum-free medium. Transwell chamber was used to evaluate the invasive ability of cells. 100 μL Matrigel, was pre-coated in the upper chamber and 500 μL serum-free medium was added to the lower chamber. Then, 100 μL cell suspension containing 1 × 10^5^ cells was added to the upper chamber. Incubated at room temperature for 18–24 h, wiped off the remaining cells in the upper chamber with cotton balls. After washing with PBS, the cells in the lower chamber were fixed and stained with 0.5% crystal violet. Under the microscope, four different visual fields were randomly selected to obtain staining images, and the number of invaded cells was counted.

### Statistical analysis

The results of at least 3 independent experiments were expressed as mean ± SD. Student *t*-test was used to analyze the data between the two groups, and SPSS 20.0 software (SPSS Inc. Chicago, IL, USA) was used for statistical analysis and *p* < 0.05 and *p* < 0.01 were considered to be statistically significant.

## Results

### Knockdown of CKS1B inhibits the survival of HCC cells

**Figure 1.**
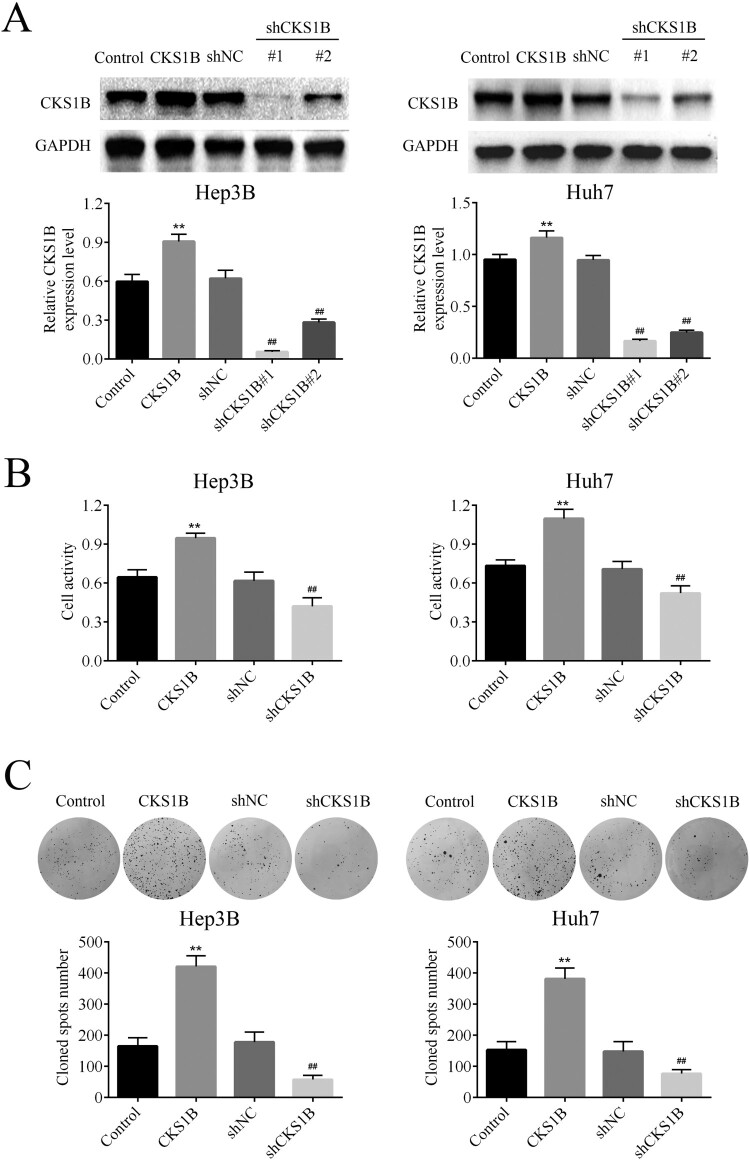
Abnormal expression of CKS1B regulates the survival of HCC cells. (A) Western blot was used to detect the protein expression of CKS1B in Hep3B and Huh7 cells after overexpression and knockdown of CKS1B. (B) MTT assay was used to detect the viability of Hep3B and Huh7 cells after overexpression and knockdown of CKS1B. (C) Colony formation assay was used to detect the proliferation of Hep3B and Huh7 cells after overexpression and knockdown of CKS1B. ** compared with Control, *p* < 0.01. ## compared with shNC, *p* < 0.01.

In order to explore whether CKS1B acts as an oncogene in HCC progression, the effect of CKS1B overexpression and knockdown on the survival of HCC cells were respectively examined. The results of western blot analysis showed that the overexpression plasmid effectively up-regulated the expression of CKS1B, while shRNA effectively down-regulated the expression of CKS1B in Hep3B and Huh7 cells. In particular, sh1# showed better knock-down effect on CKS1B, which was thus selected for follow-up experiments (Figure 1(A)). The viability of Hep3B and Huh7 cells was detected by MTT, and it was found that overexpression of CKS1B significantly enhanced the viability of HCC cells, but CKS1B knockdown significantly decreased the viability of HCC cells (Figure 1(B)). Furthermore, the colony formation assay was carried out to explore the effect of CKS1B on the colony formation of HCC cells. The results showed that overexpression of CKS1B significantly enhanced the colony formation of HCC cells, but knockdown of CKS1B showed the reversed results (Figure 1(C)). These results confirmed that knockdown of CKS1B had an inhibitory effect on the survival of HCC cells.

### Knockdown of CKS1B promotes the apoptosis in HCC cells

**Figure 2.**
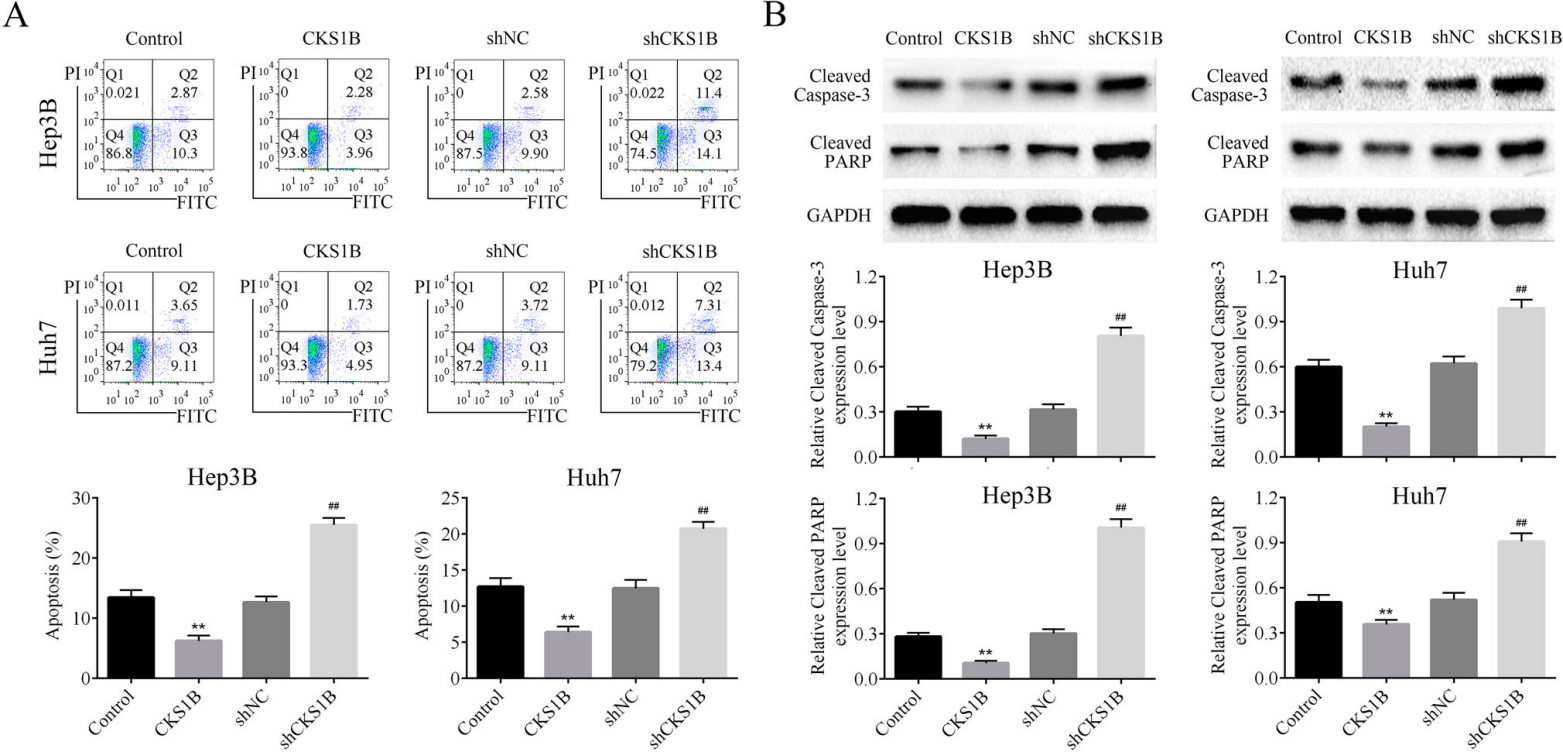
Abnormal expression of CKS1B regulates the apoptosis of HCC cells. (A) The apoptotic percentage of Hep3B and Huh7 cells after overexpression and knockdown of CKS1B was detected by flow cytometry. (B) Western blot analysis on the expression of Cleaved Caspase3 and Cleaved PARP in Hep3B and Huh7 cells after overexpression and knockdown of CKS1B. ** compared with Control, *p* < 0.01. ## compared with shNC, *p* < 0.01.

The effects of abnormal expression of CKS1B on the apoptotic rate of Hep3B and Huh7 cell lines were then detected by flow cytometry. CKS1Boverexpression inhibited the apoptosis of HCC cells, whereas knockdown of CKS1B significantly increased the apoptotic rate of HCC cells (Figure 2(A)). The levels of apoptosis-related proteins Cleaved Caspase-3 and Cleaved PARP in HCC cells were also examined by Western blot analysis. The results showed that CKS1B overexpression significantly decreased the expression of Cleaved Caspase-3 and Cleaved PARP, while knockdown of CKS1B significantly increased the expression of Cleaved Caspase-3 and Cleaved PARP in HCC cells (Figure 2(B)). These results confirmed that knockdown of CKS1B promoted the apoptosis of HCC cells.

### Knockdown of CKS1B inhibits the migration and invasion of HCC cells

**Figure 3.**
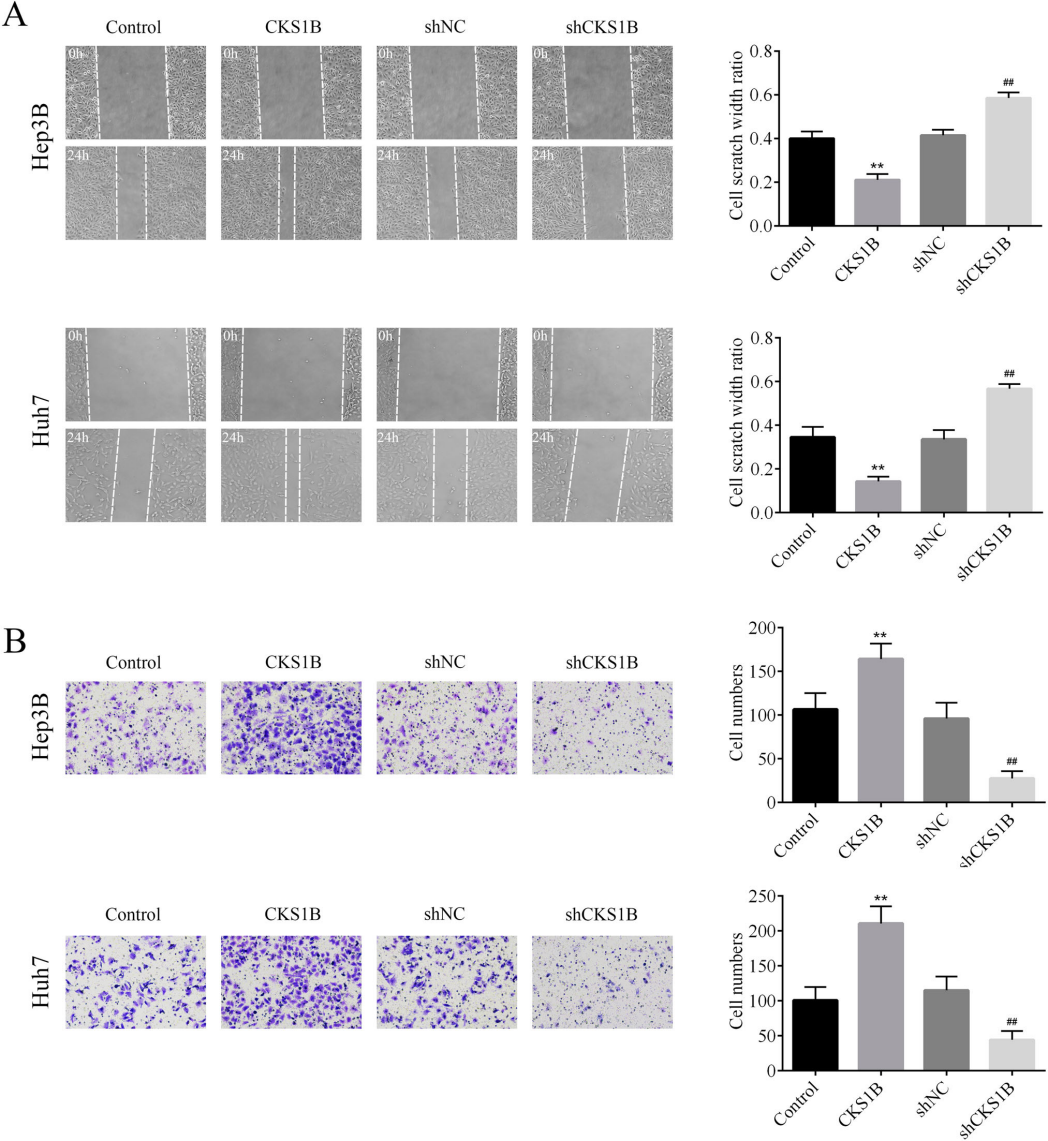
Abnormal expression of CKS1B regulates the migration and invasion of HCC cells. (A) The migratory abilities of Hep3B and Huh7 cells after overexpression and knockdown of CKS1B was observed by the wound healing experiment. (B) The invasive abilities of Hep3B and Huh7 cells after overexpression of CKS1B and knockdown of Huh7 was observed by the Transwell assay. ** compared with Control, *p* < 0.01. ## compared with shNC, *p* < 0.01.

The effects of abnormal expression of CKS1B on the migration and invasion of Hep3B and Huh7 cells were then examined. The results of wound healing assay showed that the scratch width ratio of CKS1B overexpression group was increased after 24 h, while the scratch width ratio of shCKS1B group was decreased after 24 h, indicating that knockdown of CKS1B inhibited the migratory ability of Hep3B and Huh7 cells (Figure 3(A)). The invasion assay showed that the invasive number of Hep3B and Huh7 cells of overexpression CKS1B group was significantly increased, while that of shCKS1B group was significantly decreased as compared to the corresponding controls (Figure 3(B)). Taken together, knockdown of CKS1B had a significant inhibitory effect on the migration and invasion of HCC cells.

### Knockdown of CKS1B affects HCC cell function through inhibition of JAK-STAT3 pathway activation

**Figure 4.**
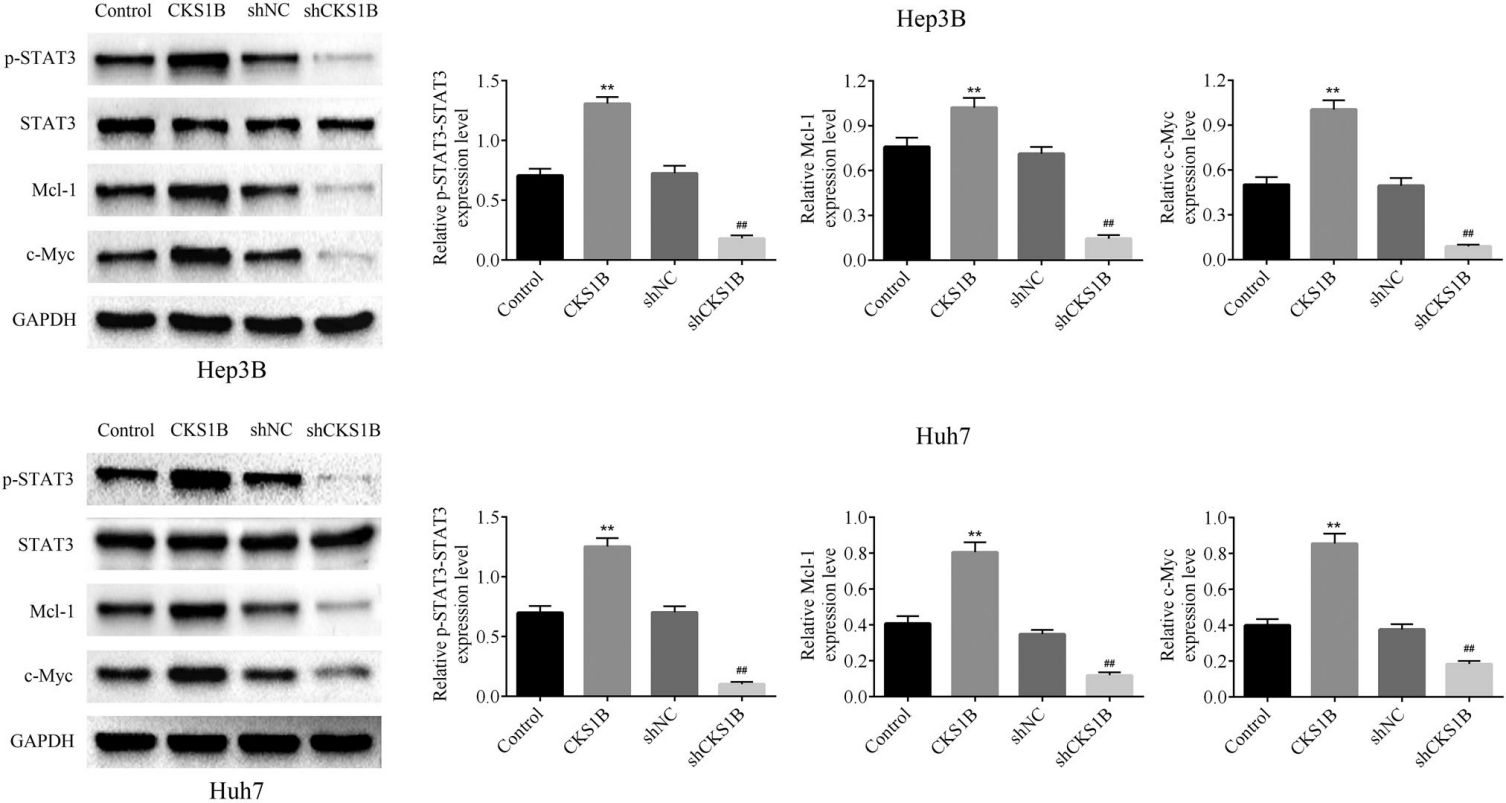
Abnormal expression of CKS1B regulates the activity of JAK/STAT3 signal pathway. Western blot was used to detect the protein expression levels of p-STAT3, STAT3, Mcl-1 and c-Myc in Hep3B and Huh7 cells after overexpression and knockdown of CKS1B. ** compared with Control, *p* < 0.01. ## compared with shNC, *p* < 0.01.

To verify whether the involvement of JAK-STAT3 signaling pathway, an important pathway associated with cancer metastasis, in the oncogenic role of CKS1B in HCC progression, the western blot analysis was used to detect the effect of abnormal expression of CKS1B on the expression of JAK-STAT3 signal pathway in Hep3B and Huh7 cells (Figure 4 and supplemental Figure S1). The results showed that CKS1B knockdown significantly inhibited the expression of p-STAT3, while had no significant regulatory effects on the expression of phosphorylated JAK1 and total JAK1 protein. It was shown that CKS1B overexpression promoted the activation of JAK/STAT3 pathway by increasing the level of p-STAT3(Figure 4(A)). In addition, knockdown of CKS1B also significantly inhibited the expression levels of STAT3 target genes Mcl-1, c-Myc, TIMP-1, Bcl-2 and VEGF. On the contrary, CKS1B overexpression caused the completely reversed results (Figure 4(B)). To sum up, these results demonstrated that the involvement of JAK-STAT3 pathway activity in the oncogenic role of CKS1B in HCC progression.

## Discussion

Chromosome loss and gain are often detected in patients with HCC, of which the amplification of chromosome 1q21-23 has been reported related to the early development of HCC(Wong et al. 1999). About 40% of patients with advanced metastatic HCC had regional 1q21-q22 gain, and 1q21 was the most frequently amplified region on chromosome 1q(Wang et al. 2002; Ma et al. 2008). This study suggested that 1q21 may contain multiple oncogenes, and the amplification of these oncogenes plays an important role in the pathogenesis of HCC. In particular, CKS1B, a gene on 1q21, has been reported to be frequently up-regulated in HCC tissues and cell lines(Lee et al. 2011), which enhanced the proliferative ability and metastatic potential of HCC cells(Kang et al. 2019). A series of functional assays in this study showed that CKS1B overexpression enhanced the viability, but decreased the apoptosis of Hep3B and Huh7 cells, confirming that CKS1B promoted the proliferation and survival of HCC cells.

HCC-related deaths are closely related to advanced distant metastasis(Bertuccio et al. 2017). Ching-Wen Huang and colleagues found that the up-regulation of CKS1B expression was related to the clinical invasiveness of HCC by data mining of Stanford University microarray database combined with the clinicopathological characteristics of patients(Huang et al. 2010). It is thus speculated that CKS1B overexpression induced metastasis in HCC and thus promoted the malignant progression. In indeed, in this study, the number of cells that underwent migration and invasion was dramatically reduced after knockdown of CKS1B, which is the first evidence confirming the inhibitory effect of CKS1B on the migration and invasion of HCC cells.

CKS1B was previously reported to promote drug resistance in myeloma cells through activation of the JAK/STAT3 signaling pathway, which revealed a link between CKS1B and JAK/STAT3 pathway (Shi et al. 2010). It is well known that JAK/STAT3 pathway is considered as one of the most promising new targets for cancer therapy due to its role in tumor cell proliferation, survival, invasion and immunosuppression(Yu et al. 2014; Johnson et al. 2018). Here, the results showed that knockdown of CKS1B significantly inhibited STAT3 phosphorylation, indicating that STAT3 is a downstream signaling pathway of CKS1B. Consistently, evidences have suggested that STAT3 activation is associated with HCC development, including cell invasion and angiogenesis, and STAT3 inhibitor S3I-201 blocks the malignant biological behaviors in HCC(Yan et al. 2017; Tu et al. 2020). This study further elucidated that CKS1B plays a regulatory role in HCC through the JAK/STAT3 pathway and regulates the expression of STAT3 target genes, including Mcl-1, c-Myc, TIMP-1, Bcl-2 and VEGF. Among them, Mcl-1 and Bcl-2 exerted anti-apoptotic effects in HCC cells (Cucarull et al. 2020), c-Myc and VEGF showed strong pro-tumor growth effects(Zhang et al. 2020), and TIMP-1 was involved in the regulation of HCC metastasis (Wu et al. 2020). Taken together, CKS1B was involved in HCC progression by influencing the expression of downstream target genes of JAK/STAT3 pathway.

This study is the first time demonstrating the potential molecular mechanism underlying the effects of CKS1B on the activation of the JAK/STAT3 signaling pathway, complementing the evidence and molecular pathways for CKS1B or the JAK/STAT3 pathway as a therapeutic target for HCC. In conclusion, this study confirms that CKS1B is an oncogene that promotes HCC progression through activation of the JAK/STAT3 signaling pathway, which may act as a new therapeutic target for HCC treatment. The exactly interactive mechanisms between CKS1B and JAK/STAT3 pathway should be confirmed by further investigations.

## Supplementary Material

Supplemental Material

## Data Availability

All data generated or analyzed during this study are included in this published article.
