# Supplementary figures and images for: CKS1B promotes the progression of hepatocellular carcinoma by activating JAK/STAT3 signal pathway
Source: Anim Cells Syst (Seoul). 2021 Jul 14;25(4):227–34. doi: 10.1080/19768354.2021.1953142 (PMC8366641; doi:10.1080/19768354.2021.1953142)

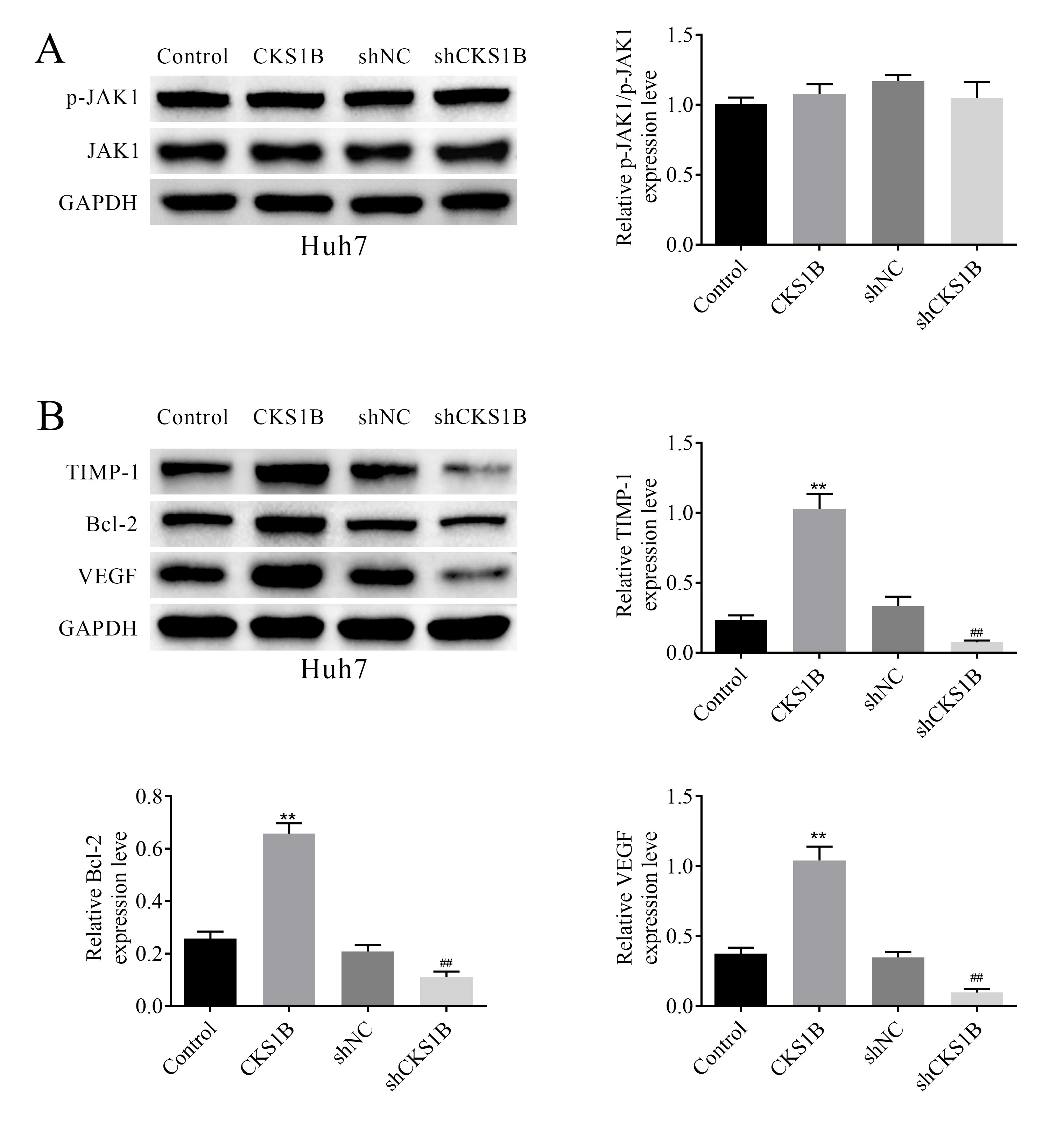

Supplement: Supplemental Material [file TACS_A_1953142_SM7134.jpg]
